# A Novel High Discriminatory Protocol for the Detection of *Borrelia afzelii*, *Borrelia burgdorferi* Sensu Stricto and *Borrelia garinii* in Ticks

**DOI:** 10.3390/pathogens11111234

**Published:** 2022-10-25

**Authors:** Giulia Chiappa, Matteo Perini, Alessandra Cafiso, Riccardo Nodari, Peter Wilhelmsson, Per-Eric Lindgren, Anna Omazic, Karin Ullman, Sara Moutailler, Petter Kjellander, Chiara Bazzocchi, Giulio Grandi

**Affiliations:** 1 Department of Veterinary Medicine and Animal Science, University of Milan, 26900 Lodi, Italy; 2 Romeo and Enrica Invernizzi Pediatric Research Center, Department of Biomedical and Clinical Sciences L. Sacco, University of Milan, 20157 Milan, Italy; 3 Department of Biomedical and Clinical Sciences, Division of Inflammation and Infection, Linköping University, 581 83 Linköping, Sweden; 4 Department of Clinical Microbiology, Region Jönköping County, 556 52 Jönköping, Sweden; 5 Department of Chemistry, Environment and Feed Hygiene, National Veterinary Institute, 751 89 Uppsala, Sweden; 6 Department of Microbiology, National Veterinary Institute, 751 89 Uppsala, Sweden; 7 ANSES, Laboratoire de Santé Animale, Ecole Nationale Vétérinaire d’Alfort, INRAE, UMR BIPAR, F-94700 Maisons-Alfort, France; 8 Grimsö Wildlife Research Station, Department of Ecology, Swedish University of Agricultural Sciences, 730 91 Riddarhyttan, Sweden; 9 Department of Biomedical Sciences and Veterinary Public Health, Swedish University of Agricultural Sciences, 751 89 Uppsala, Sweden

**Keywords:** *Borrelia burgdorferi* sensu lato, molecular typing, real time-PCR, *Ixodes*

## Abstract

Bacteria of the *Borrelia burgdorferi* sensu lato complex are the causative agents of Lyme borreliosis (LB). Even if the conventional diagnosis of LB does not rely on the species itself, an accurate species identification within the complex will provide a deepened epidemiological scenario, a better diagnosis leading to a more targeted therapeutic approach, as well as promote the general public’s awareness. A comparative genomics approach based on the 210 *Borrelia* spp. genomes available in 2019 were used to set up three species-specific PCR protocols, able to detect and provide species typing of *Borrelia afzelii*, *Borrelia burgdorferi* sensu stricto (s.s.) and *Borrelia garinii*, the three most common and important human pathogenic Lyme *Borrelia* species in Europe. The species-specificity of these protocols was confirmed on previously identified *B. afzelii*, *B. burgdorferi* s.s. and *B. garinii* specimens detected in *Ixodes ricinus* samples. In addition, the protocols were validated on 120 DNA samples from ticks collected in Sweden, showing 88% accuracy, 100% precision, 72% sensitivity and 100% specificity. The proposed approach represents an innovative tool in epidemiological studies focused on *B. burgdorferi* s.l. occurrence in ticks, and future studies could suggest its helpfulness in routine diagnostic tests for health care.

## 1. Introduction

Lyme borreliosis (LB), one of the most common tick-borne diseases in Europe, the United States (US) and Asia, is a multisystemic infectious and inflammatory disease caused by spirochetes of the *Borrelia burgdorferi* sensu lato (s.l.) complex transmitted by hard ticks belonging to the family Ixodidae [1,2,3,4]. In recent decades, the incidence of LB in humans has increased by approximately 200% worldwide, resulting in between 240,000 to 440,000 new cases per year in the US [5] and about 86,000 human LB cases in Europe [6,7]. Most LB European cases are reported from Eastern Europe, Austria, Germany, Scandinavia and Slovenia [8]. However, LB is not a notifiable disease in all studied countries, for example, in Sweden, due, among several reasons, to the considerable variations regarding the availability and quality of collected data [9].

Within the *B. burgdorferi* s.l. complex, the most widespread and common species causing LB in Europe are *Borrelia afzelii*, *Borrelia garinii* and *Borrelia burgdorferi* sensu stricto (s.s.), the latter is also widespread in the US [10]. The main vectors of these bacteria in Europe are ticks of the genus *Ixodes*, and in particular, *Ixodes ricinus*. Several factors are currently affecting the geographical range and the increasing abundance of ticks in Europe. *Ixodes ricinus* has, for example, expanded its range in northern Sweden and has become more abundant in central and southern Sweden during the last three decades [11]. This phenomenon could be attributed to a warmer climate with milder winters. Indeed, a prolonged growing season permits greater survival and proliferation over a larger geographical area of both the tick itself and tick maintenance hosts [11].

LB symptoms in humans can vary depending on the different species involved in the infection. In particular, *B. afzelii* has a predilection for causing skin manifestations, *B. garinii* for the development of neuroborreliosis, and *B. burgdorferi* s.s. for arthritis symptoms [12,13,14,15,16]; however, each species carries the potential to affect any other organ system. Therefore, the accurate and specific identification of strains belonging to the *B. burgdorferi* s.l. complex results are of great importance for the correct diagnosis and therapeutic approach in the vertebrate host. At the same time, the correct evaluation of which species is present in ticks of a certain area is pivotal to studying the epidemiology and ecology of *B. burgdorferi* s.l. Additionally, this would provide a better risk assessment and promote awareness against tick exposure [16,17,18].

However, LB is often reported to occur with non-specific symptoms (i.e., fatigue, headache, stiffness, fever [19]), resulting in persistent infection and sometimes in chronic disease (i.e., acrodermatitis chronica atrophicans or chronic Lyme arthritis [20]).

*Borrelia* genome is composed of a linear chromosome of 910 kilo-base pairs (kbp) and linear and circular plasmids of over 600 kbp, with several recombination events currently described [21]. The diverse clinical pictures and the lack of LB-specific symptoms could thus be related to the presence of pathogenicity and virulence genes found in plasmids. It is indeed known that genetic material associated with both chromosomes and plasmids can be subjected to gene transfer, duplication and loss, leading to differences at both inter- and intra-species levels [20,22].

Due to this genome complexity, several molecular approaches for the identification of *B. burgdorferi* s.l. bacteria at the species level have been developed. Some of these methods include qualitative PCR protocols based on multi-locus sequence typing (MLST; e.g., [23]), nested PCR (e.g., [24,25,26]), amplicon sequencing for species determination (e.g., [27]), and qPCR systems based on species-specific probes [28,29,30,31]. However, the described approaches are often characterized by low sensitivity and/or low specificity, leading to possible false negative or false positive results [15,32,33]. Additionally, many of these analyses are time-consuming and expensive, especially when a high number of samples need to be tested. A novel approach, based on a microfluidic BioMark™ dynamic array system named Fluidigm, was introduced by Michelet et al. [34]. The tool can potentially amplify simultaneously several different PCR targets belonging to multiple tick-borne pathogens (e.g., *Rickettsia* spp., *Anaplasma* spp., *Borrelia* spp., viruses). This method was, for example, applied aiming to study the occurrence of tick-borne pathogens in southern Scandinavia [35,36]. Concerning *Borrelia* spp., the Fluidigm system provides a concurrent PCR amplification for the *Borrelia* genus and eight *Borrelia* species (*B. afzelii*, *B. bissetti*, *B. burgdorferi* s.s., *B. garinii*, *B. lusitaniae*, *B. miyamotoi*, *B. spielmanii* and *B. valaisiana*). The sensitivity of the system is improved by a further PCR-based pre-amplification performed before loading the sample on the chip. However, Fluidigm can be performed only with specific disposables and dedicated instruments.

The aim of this study was to develop an easy, fast and reliable real-time PCR tool, based on the amplification of specific gene fragments of the main LB etiological agents in Europe (*B. afzelii*, *B. burgdorferi* s.s. and *B. garinii*), to better comprehend the distribution of these species and raise awareness of clinicians and the general public.

The unique fragments identified for each species of interest were determined following a comparative genomics approach based on the available *Borrelia* spp. genomes. The proposed protocol was validated on DNA samples from ticks collected in Sweden.

## 2. Materials and Methods

### 2.1. Genomes Download and Annotation Revision

*Borrelia* genome files (n = 210) were downloaded from the PATRIC database [37] and accessed on 16 July 2019. The assemblies were analyzed with OrthoANI [38] to assess their average nucleotide identity (ANI): genomes with ANI > 95% were assigned to the same cluster as described by Jain et al. [39]. Each group was named after the most abundant species in the respective cluster. This allowed us to find any mistakes and confirm the correct annotation of the genome’s assemblies.

### 2.2. Target Genes Selection

The pan-genome of *Borrelia* (i.e., the set of all the genes/Open Reading Frames, ORFs, in the genus) was calculated, by analyzing the downloaded genomic sequences with Roary software version 3.11.2 [40]. Briefly, the tool takes assembly genomes as an input, and the ORFs are called and clustered based on their genetic similarity allowing to group the sequences in orthologous clusters representative of the genes present in the dataset. A principal coordinate analysis (PCoA) was performed based on the gene presence/absence in the genomes with the R package Adegenet [41]. PCoA represents, in a Cartesian space, the patterns found in distance matrices to explain most of the variance in the data set. Therefore, PCoA analysis characterizes the degree of similarity of a set of genomes, considering the whole information derived from the gene presence/absence analysis. Subsequently, based on the PCoA results, the clusters of species were identified by the discriminant analysis of principal components (DAPC [42]), and the contribution of each gene to the discrimination of a specific cluster on the PCoA, was determined by the “loading plot” function of the package Adegenet [41]. Briefly, using this approach, higher loading scores are attributed to genes that have the largest between-species variance and the smallest within-species variance. The genes with the highest loading scores were selected for *B. afzelii*, *B. burgdorferi* s.s. and *B. garinii*. The selected genes were chosen to be targets for the newly designed real-time PCR amplification (qPCR).

### 2.3. Primer Design

The sequences of the selected genes were analyzed using EasyPrimer [43] to identify the most suitable regions for primer design, and then species-specific primers were manually designed. For each species, the primers and the specificity of the amplified fragments were validated in silico by BLAST searches [44]. In addition, the cross-reaction between these primers and various organisms (e.g., mammals_taxid: 40,674; hard ticks_taxid: 6939; *Anaplasmataceae* spp_taxid: 942) were also tested by BLAST searches [44] excluding *B. burgdorferi* s.l._taxid: 64,895 from the analyses.

For each primer set, the absence of homopolymeric DNA tracts was assessed and the annealing temperature was calculated using the “Oligo Analysis Tool” (https://eurofinsgenomics.eu/en/ecom/tools/oligo-analysis/ (accessed on 1 February 2020), Eurofins Genomics Vimodrone, Italy).

### 2.4. Real-Time PCR Assay Set Up

The three species-specific primer sets for the typing protocols were used in qPCR by the CFX Connect Real-time PCR detection system (Biorad^®^, Hercules, CA, USA). Each 20 µL reaction contained a final concentration of 1× SsoAdvanced Universal SYBR^®^ Green Supermix (Biorad^®^, Hercules, CA, USA), 0.25 µM of each primer, 1 µL of tick DNA and ddH_2_O up to the final volume. The thermal profile for the three reactions was set up as follows: 95 °C for 180 s; 40 cycles (95 °C for 10 s, 52 °C for 15 s and 72 °C for 15 s) and a melt curve from 55 °C to 95 °C with increments of 0.5 °C per cycle. Each sample was tested in duplicate.

### 2.5. Protocol Validation

A preliminary validation to test the species-specificity of the newly designed primers was performed on the nucleic acids obtained from six *B. burgdorferi* s.l.-positive female *I. ricinus* samples (two *B. afzelii* strains, two *B. burgdorferi* s.s. strains and two *B. garinii* strains; see Table S1) identified by the amplification and sequencing of a 5S-23S rRNA fragment of the spirochetes [26]. Briefly, total nucleic acid (NA) was extracted with MagNA Pure LC Total Nucleic Acid Isolation Kit (Roche, Basilea, Swiss) and reverse-transcribed to cDNA using illustra™ Ready-to-Go RT-PCR Beads kit (GE Healthcare, Little Chalfont, UK) as described in [45]. Each sample was tested with the three newly designed primer sets in separate tubes. To evaluate the accuracy, precision, sensitivity and specificity of the protocol, a much larger dataset of ticks (n = 120) was tested with the three designed PCR primer sets. In detail, the total NA was extracted from the ticks using the Magnatrix 8000+ extraction robot (Magnetic Biosolutions, Stockholm, Sweden) and the Vet Viral NA kit (NorDiag ASA, Oslo, Norway) and reverse-transcribed to cDNA using illustra™ Ready-to-Go RT-PCR Beads kit (GE Healthcare, Little Chalfont, UK). These samples were previously tested both for DNA and RNA-viral pathogens by Fluidigm (unpublished data). For that analysis, a mix of equal parts of total synthesized NA and cDNA were used as templates.

The 120 samples included 62 NA samples retrieved from the RåFäst-project collection (i.e., questing ticks collected by cloth-dragging method from Grimsö and Bogesund, Sweden, in 2013 [46]; Table S2) and 58 NA samples retrieved from the CLINF-project collection (i.e., ticks detached from dogs or cats in northern Sweden during 2018–2019). All the NA samples were stored at −20 °C at the National Veterinary Institute (Uppsala, Sweden; Table S2).

Additionally, samples with incoherent typing results between Fluidigm and the newly described protocol were also subjected to the amplification and sequencing of a 5S-23S rRNA fragment, as described by Wilhelmsson et al. [26]. Metrics to evaluate the new protocols were computed considering the adjustments made to the Fluidigm typing after the 5S-23S rRNA approach of the selected samples with incoherent results. Accuracy ((true positive + true negative)/total samples)), precision (true positive/(true positive+ false positive)), sensitivity (true positive/(true positive + false negative)) and specificity (true negative/(true negative + false positive)) were calculated.

## 3. Results

### 3.1. Bioinformatics Results: Validating PATRIC Annotation

**Figure 1:**
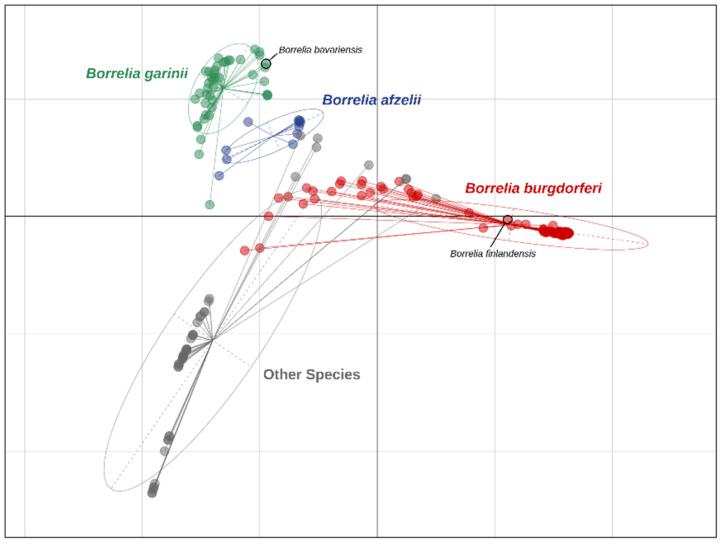
Principal coordinate analysis (PCoA) plot of the *Borrelia* genomes performed on the gene presence/absence analysis. Each dot represents a genome. The colors were manually added to group the genomes based on the average nucleotide identity (ANI) analyses: the genomes with an ANI > 95% were clustered and highlighted by the same color. Moreover, two genomes were explicitly indicated in the plot because their annotation on the PATRIC database (in black) was incoherent with the ANI clusters computed in this work (indicated by the color of the dot).

Average nucleotide identity clusters of the 210 downloaded *Borrelia* genomes revealed that 10 out of 210 (4.8%) were mis-annotated. A table with the correspondence of the genome annotation with the clusters of genomes with ANI > 95% is available in Table S3. PCoA performed on the gene presence/absence analysis is reported in Figure 1, where two out of the 10 mis-annotated genomes are highlighted (*B. burgdorferi* s.s., annotated in PATRIC as *Borrelia finlandensis*—GCF_000181875.2—and *B. garinii,* annotated in PATRIC as *Borrelia bavariensis*—GCF_003814425). After these corrections, the final composition of the database was: 11 *B. afzelii* genomes, 112 *B. burgdorferi* s.s. genomes, 43 *B. garinii* genomes and 44 genomes from other *Borrelia* species.

### 3.2. Bioinformatics Results: Target Genes Selection and Primers Design

Pangenome analysis revealed the absence of core genes among the 210 analyzed genomes (i.e., the absence of a set of orthologous sequences conserved in all aligned genomes), and DAPC analysis allowed the identification of species-specific genes. The three primer pairs designed on the genes selected to be highly specific and discriminant for each of the three *B. burgdorferi* s.l. species of interest are reported in Table 1.

The genes selected on the *B. burgdorferi* s.l. chromosome were annotated as follows:

*B. afzelii*: tRNA (adenosine(37)-N6)-threonylcarbamoyltransferase complex dimerization subunit type 1 TsaB [*Borrelia afzelii*] Protein Sequence ID: WP_004790520.1

*B. burgdorferi* s.s.: exodeoxyribonuclease III [*Borrelia burgdorferi*] Protein Sequence ID: WP_002656039.1

*B. garinii*: ribosome maturation factor RimP [*Borrelia garinii*] Protein Sequence ID: WP_029362206.1

### 3.3. Protocol Validation

The results of the three qPCRs carried out on the control samples obtained from six ticks (two positive for *B. afzelii*, two for *B. burgdorferi* s.s. and two for *B. garinii*; see Table S1), confirmed the primers species-specificity and the absence of cross-amplification among the three species. The specificity of each reaction was confirmed through the sequencing of the amplified fragments that showed 100% identity with the fragment of the corresponding species. The newly designed protocol was called LyDet, as it is aimed at Lyme bacteria detection. The NA previously extracted from the larger dataset of 120 tick specimens was typed by LyDet, and the results were compared to those obtained by Fluidigm on the same dataset. LyDet protocol highlighted the presence of 12 *B. afzelii* out of 24 Fluidigm-positives, 1 *B. burgdorferi* s.s. out of 9 Fluidigm-positives and 19 *B. garinii* out of 22 Fluidigm-positives. Additionally, six samples were identified by Fluidigm as *Borrelia* spp. (n = 4), *B. spielmanii* (n = 1) and *B. miyamotoi* (n = 1) were identified as *B. garinii* (n = 4) and *B. afzelii* (n = 2) by LyDet; these results were also confirmed by 5S-23S rRNA amplicon sequencing (Table S2). In addition, the eight samples typed as *B. burgdorferi* s.s. by Fluidigm and negative to LyDet protocol also resulted as being negative to the 5S-23S rRNA amplification (Table S2). Lastly, all the *Borrelia*-negative samples to Fluidigm were also negative by LyDet.

A comparative matrix of the results of both Fluidigm and LyDet methods is reported in Table 2. In detail, the matrix compares the two typing approaches on the 120 samples considering the differences between the two methods (LyDet and Fluidigm). LyDet showed 88% accuracy, 100% precision, 72% sensitivity and 100% specificity.

## 4. Discussion

The comparative genomics performed in this work highlights the lack of a core genome in *Borrelia* genus. This feature makes it difficult to identify the proper target gene to develop an unequivocal molecular typing protocol in general for *B. burgdorferi* s.l. complex species, and in particular for the three main etiological agents of LB in Europe (*B. afzelii*, *B. burgdorferi* s.s. and *B. garinii*). Indeed, numerous typing methods to identify *Borrelia* spp. that cause LB in both arthropods and vertebrates have been proposed over the years [47].

Thus, the best approach for *B. burgdorferi* s.l. species typing should be Whole Genome Sequencing (WGS), although several issues related to genome assembling are still limiting factors [48]. However, this technique may still be time-consuming, requires complex data computing and is still expensive to apply in routine diagnostics or in epidemiological studies.

Interestingly, comparative genomics on several *Borrelia* spp. has revealed that frequent inaccurate species assignments are present in public databases. This can possibly lead to incorrect interpretations concerning, e.g., the circulation of a certain *B. burgdorferi* s.l. species in a specific geographical area (leading to non-reliable distribution patterns), or incorrect diagnosis could also occur, resulting in improper therapeutic strategies. To our knowledge, there is no standardized procedure to fill the gaps in these discrepancies, and this can represent a growing problem in the future.

The aim of this work was to develop a typing protocol for an easier, rapid detection and identification of *B. afzelii*, *B. burgdorferi* s.s. and *B. garinii* in ticks. For this purpose, bioinformatics analyses were performed to select species-specific loci for each species. Based on the alignment of each locus, species-specific primers were designed on conserved regions flanking variable ones. The intraspecific variability of the amplified fragments would not have allowed the specificity—and consequently the sensitivity—of a probe-based approach. For this reason, the three newly designed qPCRs were set up in separate tubes and using SybrGreen reagent as a fluorescent molecule. The species-specificity of the protocol and the absence of cross-reactions with other *Borrelia* species were assessed on a dataset of already species-typed samples identified as *B. afzelii*, *B. burgdorferi* s.s. and *B. garinii,* as well as on tick samples previously analyzed and typed using a Fluidigm approach (unpublished data). Furthermore, two specimens identified using Fluidigm as *B. miyamotoi* and *B. spielmanii* were amplified by LyDet assay and assigned to *B. afzelii* and *B. garinii* species, respectively. The subsequent sequencing of the 5S-23S gene fragment confirmed the result provided by LyDet, reiterating the species-specificity of the proposed protocol.

Some previously published investigations on *B. burgdorferi* s.l. in ticks revealed that species typing was occasionally not determinable [26,34]. On the contrary, the high sensitivity of LyDet protocol allowed the typing of three *B. garinii* and one *B. afzelii,* which were generically assigned to *Borrelia* spp. group by Fluidigm, thus leading to an improvement of the species-specific detection of *Borrelia* bacteria.

There are few studies published that quantify the frequency of co-infections by different *Borrelia* spp. in ticks [49,50]. This can, in part, be due to the fact that it might be unfeasible to obtain the species identification by Sanger or whole genome sequencing in samples where multiple *Borrelia* species are present. The detection of co-infections by different Lyme *Borrelia* species is another potential outcome linked to using the LyDet protocol. One potential limitation of LyDet is that this protocol is able to detect only three given *Borrelia* species, and it might thus miss other, new, emerging or re-emerging ones. This might become an issue since these undetected species could be involved in the clinical picture or could be relevant when performing a general screening aimed at assessing the occurrence of more *Borrelia* species. One possible solution would be to perform a general *Borrelia* spp. screening according to an already described method (e.g., [26]), along with the species-specific LyDet approach.

The LyDet approach confirmed the Fluidigm results to only 50% of *B. afzelii*, 11% of *B. burgdorferi* s.s. and 86% of *B. garinii* samples, while the remaining were negative. The lower detection rate of LyDet compared to Fluidigm could be attributed to the template sample. Indeed, in LyDet assays total NA alone was used as template, while in Fluidigm a mix of total NA and cDNA was used for the detection of genetic material from pathogens in the screened tick samples (i.e., DNA from bacteria and/or protozoa, as well as RNA from RNA-viruses). It is worth mentioning that the Fluidigm approach is also based on a pre-amplification step that can increase the signal of those targets showing extremely low DNA concentrations.

Even if setting up a method for the quantification of bacterial DNA (and therefore the number of bacteria) in a given sample was beyond the scope of the present study, bacterial load in ticks is considered relevant by some authors. For example, if the analyzed tick has been detached from a patient, information on the bacterial load can help in predicting transmission risks of the bacteria to the host [26,28]. In such cases, the LyDet protocol could be optimized and improved in order to quantify the species-specific bacteria load, either by using a certain amount of target sequences (i.e., through the use of plasmids) or by assessing the number of copies of the target genes of the three PCR systems in the respective *Borrelia* genomes.

Eight out of nine samples identified as *B. burgdorferi* s.s. with the Fluidigm test produced negative results when analyzed with the LyDet protocol. This result was also confirmed by the absence of amplification of the 5S-23S rRNA fragment. This can be attributed to either a low DNA quality of the template or to an interpretation of Fluidigm results for *B. burgdorferi* s.s. that “should be interpreted with care” as stated in the first description of the method [34]. However, the limited number of *B. burgdorferi* s.s. specimens detected by LyDet are coherent with low prevalence rates of this *Borrelia* species in Sweden, as recently described [26]. Further analyses should be performed on *B. burgdorferi* s.s. positive samples for an enhanced validation of the method.

The absence of gene sequencing and the sensibility and specificity of the LyDet assay make this method particularly reliable for large screenings of ticks for *B. afzelii*, *B. burgdorferi* s.s. and *B. garinii* detection. The epidemiology and ecology of these species are indeed pivotal for human health and could help to promote prevention against tick exposure. However, the low number of genomes available in PATRIC for *B. garinii* and *B. afzelii* and the genomic plasticity of *Borrelia* DNA may decrease the sensitivity of the tool. Nevertheless, genomes of *B. burgdorferi* s.l. available in the PATRIC database are continuously upgraded [37], and the addition of new genomes to our analysis would greatly benefit the specificity of the method.

Future applications of the recently developed method could include clinical investigations on LB patients to provide a quick and straightforward identification of the related *Borrelia* spp. This method could certainly complement, but not replace, the clinical evaluation and the diagnostic tests currently used. In fact, the validity of methods such as LyDet needs to be checked periodically by comparing the target sequences to those that are continuously generated and successively made available in sequence databases.

## Figures and Tables

**Table 1:** Primers designed on the ORFs found by Roary analyses and selected to be species-specific for *Borrelia afzelii*, *Borrelia burgdorferi* s.s. and *Borrelia garinii*. Sequences, annealing temperatures and fragment lengths are reported.

Primers	Sequence (5’–3’)	Tm Oligo (°C)	Amplicon Length (bp)
*Bafzelii*_qPCR_F	ATTCTTGTGGTCCTGGTT	51.4	263
*Bafzelii*_qPCR_R	TGAATCAATCTGCCCTAG	51.4	
*Bafzelii*_qPCR_F	ATTCTTGTGGTCCTGGTT	51.4	263
*Bafzelii*_qPCR_R	TGAATCAATCTGCCCTAG	51.4	
*Bb*ss_qPCR_F	TGTATTCAAGAAACTAAAGCC	52.0	128
*Bb*ss_qPCR_R	GCTCAACTTTTGAATAAATGC	52.0	
*Bgarinii*_qPCR_F	AAAAAGTGATAGAGAGTTCC	51.1	75
*Bgarinii*_qPCR_R	CCCTCTTCAAATTCATTGTC	53.2	

bp, base pairs.

**Table 2:** The comparative matrix between Fluidigm method and LyDet protocols.

			LyDet			
			*B. afzelii*	*B. burgdorferi* s.s.	*B. garinii*	NEG
		Total (120)	**14**	**1**	**23**	**82**
**Fluidigm**	*B. miyamotoi*	**9**	**1**	0	0	8
	*B. spielmanii*	**10**	0	0	**1**	9
	*B. valasiana*	**3**	0	0	0	3
	*B. afzelii*	**24**	12	0	0	12
	*B. burgdorferi* s.s.	**9**	0	1	0	**8**
	*B. garinii*	**22**	0	0	19	3
	*Borrelia spp.*	**22**	**1**	0	**3**	18
	NEG	**21**	0	0	0	21

In bold black font are reported the total number of samples identified at species level with each method. A fragment of the 5S-23S rRNA was amplified/sequenced from 14 samples, showing incoherent results between the two methods (reported in bold red font).

## Data Availability

Not applicable.

## Supplementary Materials

The following supporting information can be downloaded at: https://www.mdpi.com/article/10.3390/pathogens11111234/s1, Table S1: Information about the six *B. burgdorferi* s.l.-positive female *I. ricinus* samples (two positive for *B. afzelii* strains, two for *B. burgdorferi* s.s. strains and two for *B. garinii* strains) used for PCR primers validation; Table S2: Information about the 120 tick samples used to validate the protocol; Table S3: Correspondence table of the PATRIC genome annotation (rows) with the cluster of genomes with ANI > 95% (columns). The 10 mis-annotation found in the PATRIC database are indicated in bold red font.
